# The Role of Polyphenol in Modulating Associated Genes in Diabetes-Induced Vascular Disorders

**DOI:** 10.3390/ijms23126396

**Published:** 2022-06-07

**Authors:** Nor Anizah Mohd Nor, Siti Balkis Budin, Satirah Zainalabidin, Juriyati Jalil, Syaifuzah Sapian, Fatin Farhana Jubaidi, Nur Najmi Mohamad Anuar

**Affiliations:** 1 Centre for Diagnostic, Therapeutic and Investigative Studies, Faculty of Health Sciences, Universiti Kebangsaan Malaysia, Kuala Lumpur 50300, Malaysia; ejamdnor@gmail.com (N.A.M.N.); balkis@ukm.edu.my (S.B.B.); syaifuzahsapian17@gmail.com (S.S.); fatinfarhanajubaidi@gmail.com (F.F.J.); 2 PICOMS International University College, Taman Batu Muda, Batu Caves, Kuala Lumpur 68100, Malaysia; 3 Programme of Biomedical Science, Centre for Toxicology and Health Risk Research, Faculty of Health Sciences, Universiti Kebangsaan Malaysia, Kuala Lumpur 50300, Malaysia; satirah@ukm.edu.my; 4 Center for Drug and Herbal Development, Faculty of Pharmacy, Universiti Kebangsaan Malaysia, Kuala Lumpur 50300, Malaysia; juriyatijalil@ukm.edu.my

**Keywords:** oxidative stress, inflammation, apoptosis, phenolic acid, flavonoid, stilbene, lignan, polyphenol, vascular

## Abstract

Diabetes-induced vascular disorder is considered one of the deadly risk factors among diabetic patients that are caused by persistent hyperglycemia that eventually leads to cardiovascular diseases. Elevated reactive oxygen species (ROS) due to high blood glucose levels activate signaling pathways such as AGE/RAGE, PKC, polyol, and hexosamine pathways. The activated signaling pathway triggers oxidative stress, inflammation, and apoptosis which later lead to vascular dysfunction induced by diabetes. Polyphenol is a bioactive compound that can be found abundantly in plants such as vegetables, fruits, whole grains, and nuts. This compound exerts therapeutic effects in alleviating diabetes-induced vascular disorder, mainly due to its potential as an anti-oxidative, anti-inflammatory, and anti-apoptotic agent. In this review, we sought to summarize the recent discovery of polyphenol treatments in modulating associated genes involved in the progression of diabetes-induced vascular disorder.

## 1. Introduction

Diabetes mellitus (DM) is a metabolic disorder with persistently high blood glucose levels, resulting from an impairment of insulin production and insulin resistance [1]. It is considered to be one of the world’s most prevalent diseases and is increasing globally. The global prevalence of DM in people aged 20 to 79 is expected to rise from 10.5% (536.6 million) in 2020 to 12.2% (783.2 million) by 2045 [2]. DM contributes to vascular diseases including microvascular complications (retinopathy, nephropathy, and neuropathy) and macrovascular complications (ischemic heart disease, stroke, and peripheral vascular disease (PAD) [3]. Therefore, DM leading to vascular disorders plays a crucial role in high rates of morbidity and mortality among DM patients [4].

DM is the main culprit that induces vascular dysfunction which results in vascular disorder by disrupting the normal homeostasis of the vessel wall [5]. At the cellular level, DM causes endothelial cells (ECs) to alter cellular homeostasis, and to increase the capillary permeability via oxidative stress, inflammatory, and apoptosis mechanisms which then progress to endothelial dysfunction [6,7]. Endothelial dysfunction is the onset leading to the progression of vascular disorders [8]. Reactive oxygen species (ROS) is the main culprit that causes oxidative stress, which exerts significant damage to various cellular components including lipids, proteins, and deoxyribonucleic acid (DNA) [9]. Oxidative stress induces dysregulated expression of various genes, which further stimulate inflammation and apoptosis mechanisms and lead to insulin secretion and signaling impairment, which then further escalates to the progression of the vascular disorder and cardiovascular disease (CVD) [4]. Therefore, it is crucial to understand targeted related genes underlying the mechanisms involved in vascular dysfunction caused by DM to find therapeutic approaches that could augment vascular dysfunction for preventing DM-induced vascular disorder.

For the past few decades, extensive efforts have been invested in studying the use of natural compounds to treat DM-induced vascular injury [10]. One of the potential candidates is polyphenols, which can be found abundantly in fruits, nuts, vegetables, and cereals. There are four main subclasses of polyphenols which include phenolic acid, flavonoid, stilbene, and lignan. They have been reported to exert therapeutic effects against various pathological conditions, such as cancer, inflammation, microbial infections, CVD, and most commonly, DM [11,12,13,14,15]. These therapeutic effects are mainly mediated through radical scavenging, antioxidant, anti-inflammatory, and anti-apoptosis effects [16,17]. Polyphenols have been proven to ameliorate the progression of DM-induced vascular disorder [18,19,20]. Since polyphenols had shown potential to alleviate vascular dysfunction [21], we sought to review the therapeutic approach of polyphenols in attenuating associated genes that underlie the mechanisms of DM-induced vascular disorder by targeting oxidative stress, inflammation, and apoptosis pathways.

## 2. Vascular Complications in Diabetes

### 2.1. Macrovascular Complications

Macrovascular complications due to hyperglycemia lead to the damage and disruption of the function of large blood arteries and the heart, which are linked to cardiovascular illnesses such as atherosclerosis, myocardial infarction, cardiomyopathy, and stroke [22]. The complications can also be caused by stenosis or blockage of coronary arteries such as the aorta, cerebral or peripheral blood vessels that eventually lead to the peripheral artery, coronary artery, and cerebrovascular disease. The persistent macrovascular complications will require lifelong medical treatment [23]. The formation of these complications due to hyperglycemia takes a long time. Early macrovascular complications involve endothelial dysfunction, the development of atherosclerotic plaque formation, and culminate in plaque rupture as well as the development of thrombus, leading to organ impairment [24].

Endothelial dysfunction is the main culprit that occurs as an early episode in atherosclerosis development, leading to vascular dysfunction, vascular disorder, and cardiovascular complications [25,26,27]. Together, DM, oxidative stress, and endothelial dysfunction contribute to the progression of vascular disorder. Oxidative damage due to overproduction of ROS is induced via high glucose fluctuations due to postprandial hyperglycemia in patients with DM. Furthermore, insulin resistance in ECs with the stimulation of phosphoinositide 3-kinase (P13K)/Akt and endothelial nitric oxide (NO) synthase (eNOS) pathway is attributed to decreased NO production and increased endothelin-1 (ET-1) secretion, promoting endothelial dysfunction [28].

Advanced glycation end products (AGEs) proteins bind with their receptors (RAGE) at endothelial cells causing alternative signaling pathways to be activated, and AGEs can interact with NO to decrease bioavailability as well. This will increase intracellular oxidants and inflammatory mediators that eventually lead to cell death due to elevated irreversibly glycated lipoproteins (AGE-LP), thus accelerating atherosclerosis progession in DM [29]. There are two classes of LP involved in the atherosclerotic process, i.e., low-density LP (LDL) with pro-atherogenic potential, and the protective anti-atherogenic high-density LP (HDL). In pathological conditions, LDLs contribute to the atherosclerotic process by accumulating in the subendothelium as extracellular modified LP, releasing pro-inflammatory mediators and promoting the recruitment of inflammatory cells, including neutrophils, lymphocytes, monocytes, and macrophages, leading to foam cell formation [30]. LP-A, which is an LDL-like particle surrounded by apolipoprotein(a), has garnered growing attention recently and is now acknowledged as a risk factor for CVD patients with type 2 DM (T2DM) [31].

Peripheral arterial disease (PAD) is the early episode that promotes atherosclerosis and thrombosis of the lower limb arteries. PAD occurs in approximately 5.56% of adults worldwide and the number keeps rising [32]. PAD is associated with leg pain that leads to decreased health performance, immobility, tissue damage, and increased risk of myocardial infarction, disability, stroke, and death [33]. PAD is difficult to diagnose especially in DM patients. As a result, in DM patients, the development of arterial inflammation due to endothelial cell dysfunction, vascular smooth muscle cells (VSMCs) dysfunction, and platelet aggregation leads to vessel wall degeneration including homeostasis disruption. Until DM is diagnosed, vascular dysfunction is proportional to the blood glucose level [23].

DM contributes to the various metabolic, structural, and biochemical changes contributing to heart failure and a high risk of CVD development. The hyperglycemia, hyperlipidemia, and insulin-resistant states in DM lead to heart failure because the coronary vasculature changes to coronary artery disease and leads to cardiac dysfunction. This condition contributes to changes in extracellular matrix remodeling and cardiac fibrosis formation, as well as disturbances in vascular function, calcium regulation, cardiac contractility, cardiac efficiency and lipoapoptosis as a response to oxidative stress, inflammation, and apoptosis [34]. Together with these changes, individuals with DM experience greater cardiac dysfunction as a result of this condition. eNOS and O-linked *N*-acetylglucosamine (O-GlcNAc) can disrupt vasodilation and lead to elevated blood pressure [35]. AGEs stimulate the reduced nicotinamide adenine dinucleotide phosphate (NADPH) oxidase activity via raising NADPH oxidase 2 (NOX2) and p22phox protein expression, which in turn enhances ROS generation as well as impairs potassium voltage channels, resulting in coronary artery vasodilation failure in high blood glucose condition in rats [36].

Systemic inflammation and arterial stiffness due to atherosclerosis cause pathologic changes in cerebral blood vessels which increase the risk factors for stroke and dementia as well as DM [37]. In hyperglycemia, pathways, including oxidative stress and apoptosis, promote ROS generation and upregulation of vasoconstrictor ET-1, which triggers vascular smooth muscle dysfunction [38].

### 2.2. Microvascular Complications

Microvascular complications, whereby arterioles in the retina, renal, and nerve are commonly impaired among T2DM patients, could lead to retinopathy, nephropathy, and neuropathy [39]. Endothelial dysfunction in microvascular complications is characterized by reduced NO bioavailability, increased oxidative stress and inflammation, abnormal angiogenesis activity, impaired endothelial repair processes, and apoptosis [40,41].

Diabetic retinopathy affects the peripheral retina as well as macula, and causes blurred vision and blindness, due to vitreous blood loss or retinal objectivity in DM conditions [42]. In pathological findings, the diabetic retinopathy causes blindness and intermittent blurry vision which is characterized by microaneurysms leading to neoangiogenesis and scar tissue in the retina [43].

Diabetic nephropathy commonly occurs in DM and is the leading cause of kidney failure globally, which is affecting up to 40% of DM patients [44]. Commonly, in long-term complications of DM, patients usually develop microvascular kidney damage and various microvascular complications [45]. The increased ROS and activation of proinflammatory mediators caused by chronic hyperglycemia lead to the progression of end-stage renal disease. This condition is characterized by an increase in albuminuria and glomerular lesions which lead to a gradual decline in the glomerular filtration rate [46]. Furthermore, intraglomerular hypertension and further kidney damage are caused by an aberrant decrease in afferent arteriole resistance and an abnormal rise in efferent arteriole resistance [47] which ultimately leads to end-stage renal disease [44]. The thickening of the glomerular and tubular basement membranes, as well as mesangial development, microaneurysms formation, extracellular matrix assembling, and glomerular and tubular cell damage, all, contribute to glomerulosclerosis and tubulointerstitial fibrosis, resulting in progressive albuminuria and decrease kidney function [48,49].

Diabetic neuropathy is the microvascular vasculopathy that affects peripheral neurons. Glucotoxicity and lipid accumulation in the basement membrane are two mechanisms that induce neuropathic damage in diabetic vasculopathy. Both of these mechanisms generate demyelination that affects motor and sensitivity neurons in the periphery [50]. However, the precise mechanisms that cause diabetic neuropathy remain unknown.

## 3. Pathophysiology of Diabetes-Induced Vascular Disorder

There is a variety of physiological disruptions which may associate DM with an increase in DM-induced vascular disorder. This includes endothelial dysfunction, VSMCs dysfunction due to stimulation of oxidative stress, inflammation, and apoptosis pathways in DM. Persistent exposure to hyperglycemia causes an increase in AGEs and RAGE, polyol pathway, protein kinase C (PKC) activation pathway, and hexosamine pathway expression by inducing the ROS production and contributing to vascular endothelial dysfunction [51]. As a result, endothelial dysfunction is recognized as a key contributor to vascular dysfunction in DM, connecting all these pathological events.

Endothelial dysfunction is the inability or failure of the vascular endothelium to undergo its normal homeostasis. Hence, endothelial dysfunction is a notable event at the early stage of the development of vascular complications in DM patients [28,52]. DM-related endothelial dysfunction precedes morphological vascular changes, which include loss of capillary endothelium and intercellular junctions, altered expression, and production of adhesion glycoproteins on ECs, stimulating the binding of inflammatory cells that trigger the transendothelial migration.

The imbalance of endothelium-derived contracting and relaxing factors due to imbalances in the production of vasoactive agents and the resulting secretory responses such as inflammation and apoptosis are initial hallmarks of endothelial dysfunction factors, contributing to vascular pathological disorder [53]. Unlike normal endothelium, when exposed to vasodilators, it induces aberrant responses. The endothelium is a main target and mediator for CVD by its strategic location between the bloodstream and vascular smooth muscle. The activated oxidative pathways will generate ROS and cause increased vasoconstriction, vascular remodeling, inflammation, and apoptosis which eventually lead to endothelial dysfunction and damage [54].

Endothelium-derived NO is potent in suppressing pro-inflammatory gene expression, cellular adhesion molecules, ET-1 generation, VSMC proliferation, as well as enhancing oxidized LP which is important for blood flow preservation [55]. Damage to ECs, as well as the decrease of NO bioavailability mediated by oxidative stress activity, is likely initiated by DM-induced vascular disorder [56].

Endothelial-dependent vasodilation has been shown to be debilitated in DM patients. Hyperglycemia may cause an increase in oxidative stress and the production of inflammatory mediators in the presence of insulin resistance. The oxidative damage activates metabolic signals that disrupt the morphology and physiology of the endothelium, vascular VSMCs, and tissues which contribute to microvascular and macrovascular complication [22].

Hyperglycemia promotes the development of AGEs over time due to non-enzymatic reaction causing a complex chain of dehydrogenation and oxidation processes. Unutilized glucose reacts with free amino groups toform an unstable compound, the Schiff base, which then undergoes an internal rearrangement to form amadori products. The amadori products degrade to reactive dicarbonyl compounds which will eventually form irreversible compounds (AGEs) via oxidation and dehydration processes. These end-products have been connected to the development of DM problems and are detected in plasma, vessel walls, and tissues. They also cause excessive collagen and extracellular matrix accumulation in the vascular wall, which can contribute to the build-up of oxidized LDL. Oxidized LDLs are more prone to oxidative stress activation, which causes endothelial impairment by stimulating inflammation and apoptosis and alterations in VSMCs. The progression of DM vascular disorder is also aggravated by VSMCs’ dysfunction. DM accelerates the change of vasculature by promoting the atherogenic event in VSMCs. Endothelium cells can also affect VSMC and metabolic signal secretion activity. The process is initiated when the endothelium is injured or damaged, causing VSMCs to become dysfunctional [54].

**Figure 1:**
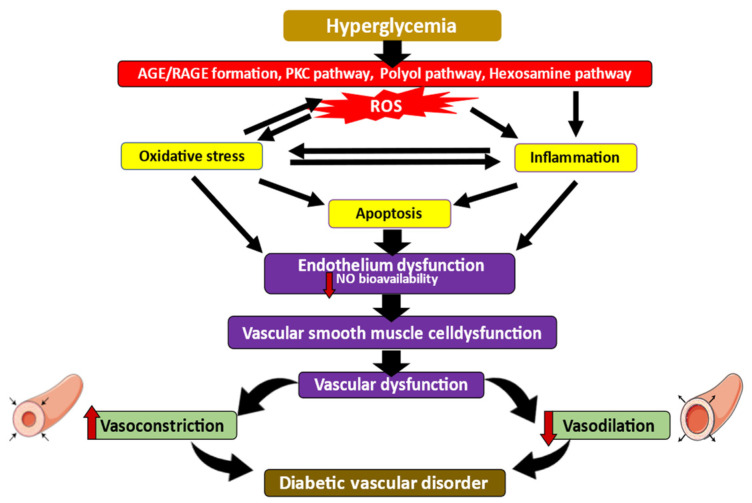
Hyperglycemia induces activation of AGE/RAGE formation, PKC pathway, polyol pathway, and hexosamine pathway and leads to ROS generation. Elevated ROS causes oxidative stress and inflammation which further trigger the apoptosis pathway. This condition will induce endothelium dysfunction by reducing the NO bioavailability. Together, all these processes will cause vascular dysfunction, resulting in diabetic vascular disorder.

A defective endothelium signals and permits macrophages and LDL to infiltrate into the medial layer of arteries to form foam cells. At this point, the vessels are ready to develop atheroma. When the macrophage-rich fatty streak forms, the VSMC migrates and proliferates into the nascent intimal lesion to form a fibrous cap [57]. This condition is the source of collagen and strengthens the atheroma, making it less likely to rupture and cause thrombosis. However, plaque, which consists of low VSMCs content, is considered as vulnerable and easily ruptured, which eventually leads to lethal thrombosis. Additionally, hyperglycemic-induced lipid modifications of LDL are regulated by increased migration and apoptosis of VSMC in atherosclerotic lesions. The LDL that has undergone non-enzymatic glycation stimulates VSMC migration in vitro, but oxidized glycated LDL can induce apoptosis of vascular SMCs [58]. Hyperglycemia also increases necrotic cell death which may contribute to diabetic vasculopathy development through hydrogen peroxide (H_2_O_2_) formation [59]. As a result, DM changes the function underlying vascular smooth muscle and causes diabetic vascular disorder. Figure 1 illustrates the pathophysiology of diabetic vascular disorder caused by hyperglycemia.

## 4. Signaling Pathways Related to Diabetes-Induced Vascular Disorder

### 4.1. Oxidative Stress

During hyperglycemic episodes, overproduction of ROS in the endothelium cells trigger vascular oxidative damage. Under normal circumstances, glucose oxidation occurs through glycolysis pathways, then proceeds to mitochondria and passes through a tricarboxylic acid (TCA) cycle to produce adenosine triphosphate (ATP) via the electron transport chain (ETC) [60]. The high level of glucose oxidation in the TCA cycle in DM causes an increase in the transfer of electrons in ETC thereby hampering ETC’s normal homeostasis and resulting in the overproduction of superoxide. Not only that, but hyperglycemia also prevents the expression of the endogenous antioxidant such as superoxide dismutase (SOD), manganese SOD (MnSOD), and uncoupling protein-1 which is responsible for scavenging ROS, which then allows elevated ROS levels and activates the alternative damaging biochemical pathways in the ECs [46]. The enhanced ROS levels in diabetic vasculature can trigger disruptions of ECs via polyol, PKC, AGE, and hexosamine pathways.

The major machinery for superoxide production in the vascular cells is NADPH oxidase with nicotinamide adenine dinucleotide (NADH) as substrate [61]. NADPH is stimulated by an elevated NADH/NAD^+^ ratio via boosting the flux through the polyol pathway [62]. Elevated levels of glucose and free fatty acid (FFA) may also augment NADH oxidase activity via PKC activation [63]. Furthermore, ROS has been produced mainly by mitochondria. The TCA cycle in mitochondria produces NADH and flavin adenine dinucleotide (FADH_2_) that act as electron donors for ETC. When intracellular glucose content rises in DM, the proton gradient rises, inhibiting electron transfer from reduced coenzyme Q to complex III of the ETC [64]. Electrons are instead transported to molecular oxygen, resulting in the formation of superoxide.

 Superoxide generation in the mitochondria also increases the intracellular generation of AGEs. AGEs affect cellular homeostasis both by affecting protein function and by stimulation of RAGE. AGEs are responsible for the increase in superoxide; just as RAGE activation boosts intracellular enzymatic production of superoxide [63]. AGE/RAGE binding also increases the activity of PKC. Uncontrolled hyperglycemia causes glucose to undergo a series of glucose reductions resulting in the stimulation of the diacylglycerol pathway (DAG) to the activation of the PKC pathway [65]. This pathway will trigger the oxidative stress mechanism by activating the NADPH oxidase enzyme which producing more ROS.

NADPH oxidase in cytosol plays a crucial role in ROS production. Activation of NADPH oxidase with the subunits’ complex (p22phox, and NOX2, p47phox, p67phox, p40phox, and activation of the small GTPase Rac) requires translocation of the cytosol to the plasma membrane, and their association with NOX2 produces ROS. The NOX family is expressed in the vessel includes NOX1, NOX2, NOX3, NOX4, and NOX5. It is well known that the NOX family is responsible for ROS production in DM [66]. The increase in activity of NADPH oxidases and uncoupled eNOS in DM, contributes to the general oxidative stress state and leads to the inflammation of vascular tissue [22].

Furthermore, NADPH is the crucial substrate for reduced glutathione (GSH) generation, a ROS scavenger. When NADPH is lowered due to hyperglycemia, the generation of GSH also will be suppressed, which further aggravates oxidative stress [67]. The escalated ROS level plays a detrimental role in oxidative stress-induced endothelial dysfunction, which further causes DM-induced vascular disorder. As a consequence, increased superoxide generation promotes the hexosamine pathway, which inhibits eNOS activation by Akt [68]. Extracellular xanthine oxidase is likely recruited by these activities, adding to the oxidative stress [69]. High blood glucose fluctuations also induce membrane translocation and stimulation of PKC by increasing the synthesis of the lipid secondary messenger DAG [67]. Activation of PKC limits the P13K pathway, restricting stimulation of Akt kinase and subsequent phosphorylation of eNOS, resulting in reduced NO generation and reducing cellular metabolism by inhibiting glucose transporter type 4 function [70]. Hyperglycemia-induced changes in Akt signaling are important in vascular pathological processes such as atherosclerosis and vascular remodeling.

The other factor that contributes to the pathogenesis of vascular disorder under high glucose concentration is due to the NO bioavailability reduction, promoting endothelial impairment. NO can scavenge free radicals. However, a pro-oxidant redox state that favors the oxidation of the eNOS cofactor tetrahydrobiopterin (BH4) can be a source for ROS generation [71]. This leads to eNOS uncoupling and the production of superoxide. In addition, superoxide generation in DM inactivates NO to form peroxynitrite (ONOO^−^). ONOO^−^ is a powerful oxidant that easily invades phospholipid membranes and induces substrate nitration. Protein nitrosylation blunts the activity of antioxidant enzymes and eNOS, contributing to eNOS uncoupling [56]. Moreover, high glucose oxidation decreases eNOS levels of vascular ECs by inhibiting the expression of hypoxia-inducible factor-1α (HIF-1α), thus reducing the NO levels [25]. The depleted NO content thus perturbs endothelial and vascular homeostasis [51].

The elevated expression of heme oxygenase 1 (HO-1) restores vascular NO production, prevents endothelial cell dysfunction, and improves vascular function. However, hyperglycemic conditions favor the downregulation of HO-1 and prompt the depletion of NO levels in vasculature. HO-1 is considered as first-line defence against oxidative damage. Hence, oxidative stress is a strong inducer of HO-1 [72]. Nevertheless, decreased expression of HO-1, as well as increased ROS, causes the responsible scavenger for oxidative stress to weaken and worsen the vascular injury. HO-1 transduction was also associated with adiponectin levels, stimulation of 5′ adenosine monophosphate-activated protein kinase (AMPK)-eNOS pathway, and improved endothelial function with diminution levels of inflammatory cytokines and free radicals [73].

Hyperglycemia additionally causes Sirtuin 1 (SIRT1) dysregulation resulting in enhanced histone 3-binding p66Sch promoter acetylation [74]. These changes induce hypomethylation of the p66Shc promoter leading to upregulation of the adaptor protein expression for glucose normalization. SIRT1 downregulation also causes increased p53 activation further promoting p66Shc gene transcription. Overexpression of p66Shc causes mitochondrial ROS accumulation leading to vascular apoptosis, vascular inflammation, and endothelial impairment [6].

Endothelial dysfunction is also associated with the AMPK stimulation. Endothelial AMPK can be activated by high glucose levels, as well as SIRT1. In contrast, protein phosphatase dephosphorylates and inactivates AMPK. In hyperglycemia, AMPK activation leads to suppressed activation of eNOS, decreased BH4 levels, and Hsp90 association with eNOS, which results in reduced NO production [75]. Another eNOS stimulation mechanism by AMPK is through the AMPK-PI3K-Akt pathway. Inhibition of AMPK activation also causes vasoconstriction in arteries through inhibition of endothelial KCa channels activity. The diminution of endothelial AMPK aggravates oxidative stress, inflammation, and apoptosis, which limits endothelial metabolism by downregulating β-oxidation of free FFAs and glycolysis [76,77]. In VSMCs, AMPK directly contracts VSMCs by increasing Ca^2+^ mechanisms, decreasing sarcoplasmic/endoplasmic reticulum calcium ATPase activity, deactivation of KATP and BKCa channels resulting in hypopolarization, and activation of Ca^2+^ entry through VOC channels under hyperglycemia [78]. By contrast, in its role in VSMCs relaxation, AMPK exerts antiproliferative, anti-migratory actions and inhibits vascular calcification [75].

### 4.2. Inflammation

Inflammation is stimulated by various factors such as oxidative stress. The formation of ROS causes elevated oxidative damage in ECs, which promotes proinflammatory cytokines and raises the expression of cellular adhesion molecules and growth factors, resulting in vascular endothelial dysfunction. Increased levels of pro-inflammatory cytokines in DM patients and experimental animals indicate that DM is associated with a persistent pro-inflammatory state [79,80]. This condition is prominently found in the onset of T2D-associated cardiovascular complications [56]. The association of inflammation and endothelial dysfunction promotes an injury-healing imbalance, which aids the evolution of microvascular and macrovascular disorders [81].

Persistent hyperglycemia is linked to a chronic proinflammatory state, which results in elevated tumor necrosis factor α (TNFα) levels by inflammatory mediators including NFκB in DM [80]. TNFα activation is induced by oxidative damage and modulates various inflammatory genes, namely IL-1β, IL-6, and monocyte chemotactic protein 1 (MCP-1) [22]. TNFα also promotes the endothelium to produce MCP-1 and cellular adhesion molecules such as intercellular adhesion molecule-1 (ICAM-1) and vascular cell adhesion molecule-1 (VCAM-1), which results in the migration of leukocytes to the endothelium’s surface and hence the development of endothelial impairment. Endothelial damage and inflammation is triggered as more leukocytes move across the endothelium. Moreover, IL-1β increases the activation of inducible NO synthase, which emits an excess of NO, thus interacting with superoxide radicals to form ONOO^−^, intensifying the inflammatory response and creating nitrosative stress [22].

Apart from that, the upregulation of NFκB worsens inflammation in the vascular by further triggering the expression of related genes, including TNFα, ET-1, interleukin IL-1, and IL-6 macrophage inflammatory protein-1 (MIP-1), VCAM-1, ICAM-1, and vascular endothelial growth factor [51,82]. Increased expression of these inflammatory factors will trigger vascular calcification, arterial stiffness, and plaque accumulation in the vessels wall of atherosclerosis [83]. A previous study by Wu et al. [84] indicated plasma inflammatory cytokine levels and expression of TNFα, IL-6, and ICAM-1 vascular ECs are significantly elevated in acute and chronic hyperglycemia in vivo DM model.

Furthermore, elevated AGEs production caused by hyperglycemia also contributes to the inflammatory episode. AGEs promote architecture changes of inflammatory proteins which then activate RAGE. The activation of AGEs/RAGE intensifies the oxidative damage in ECs, VSMCs, and inflammatory cells, which are a factor in NOX-induced ROS production, which further activates NFκB [51]. The RAGE stimulation triggers IκB kinase (IĸKβ) activation and facilitates phosphorylation-mediated proteasomal degradation of inhibitors of NFκB (Iκβ) proteins, allowing liberation of NFκB.

AGEs/RAGE interaction also enhances the oxidation of LDL [83]. RAGE attaches to oxidized LDL, which acts as a ligand affecting downstream genes including PKC activation, mitogen-activated protein kinase (MAPK), Ras-mediated extracellular signal-regulated kinase (ERK1/2), p38, stress-activated protein kinase/c-Jun N-terminal kinase (SAPK/JNK), and Janus kinase signal transducer and activator of a transcription (JAK/STAT) pathway that activates STAT3, NFκB, AP-1 and HIF-1α [85,86,87]. FFA can initiate the pro-inflammatory pathways JNK and NFκB, leading to greater secretion of TNFα and IL-6. The activation of MAPK and NFκB signaling leads to gene transcription regulation and the production of pro-inflammatory cytokines, as well as the suppression of NO production and the reduction of insulin-stimulated eNOS expression [51].

In DM, JNK activation generates phosphorylation of insulin receptor substrate (IRS-1) at serine residues, therefore causing insulin signal transduction to be downregulated and insulin resistance to occur, hence activating NFκB by impaired enzymatic activity in the phosphatidylinositol 3-kinase/protein kinase B (PI3K/Akt) pathway. JNK activation causes NFκB translocation to the nucleus, which upregulates the production and release of TNFα, IL-1, and IL-6, further increasing insulin resistance and accelerating plaque deposition in blood vessels [88].

Under high blood glucose conditions, the decrease in HO-1 expression in ECs prompts an increase in expression of VCAM-1 and MCP-1. When HO-1 is activated, macrophages in blood circulation are driven to the area of the injury, adhere to the ECs, and transendothelial migration, where they differentiate into macrophages. In atherosclerotic lesions, HO-1 is increased in macrophages as inflammation develops. Furthermore, various macrophage subtypes have recently been identified in which HO-1 expression may play a role in modifying their role in DM-induced vascular disorder [89]. Ultimately, chronic vascular inflammation causes an increase in apoptotic cells, extracellular matrix remodeling, elastic lamella breakdown, and endothelial dysfunction in atheroprone arteries, accelerating the development of DM-induced vascular disorder [90].

### 4.3. Apoptosis

Apoptosis is a natural cell death phenomenon that is required for multicellular organisms’ development and normal homeostasis [91]. Apoptosis is triggered by an increase in ROS caused by hyperglycemia, which promotes the development of oxidative damage to the DNA histones and changes DNA repair enzyme expression. High glucose levels cause a variety of cellular responses, but they all lead to functional alterations and often apoptosis. Furthermore, ROS promotes endothelial cell apoptosis and NADPH oxidase activation which trigger caspase cell death activation as shown in in vitro studies [92]. The chronic hyperglycemic state induces elevated ROS by increasing AGEs activation and interacting with the cellular receptors to activate the transcription factor such as NFκB and PKC pathways leading to cellular cell death in diabetic vascular disorder [48].

A high glucose level induces activation of proteins implicated in apoptotic cell death and including the caspase members and Bcl-2 families. Oxidative damage and inflammation stimulate the procaspases and activate caspases by proteolytic cascade which promotes cell apoptosis [93]. There are two major pathways that induce cell apoptosis including extrinsic and intrinsic pathways, and both can occur concurrently. The extrinsic pathway is characterized by the binding of a stressor to a cell’s receptor such as the binding of TNFα and Fas ligands. Their binding activates caspase-8 and caspase-9 which in turn activates the executioner caspases such as caspase-3, caspase-6, and caspase-7, resulting in the initiation of apoptosis and the progression of vascular dysfunction and various CVD.

Extrinsic and intrinsic pathways lead to cell apoptosis, and both can occur simultaneously. The binding of a stressor to a cell’s receptor in the extrinsic pathway is also known as the death receptor pathway. TNFα and Fas ligands are examples of cell death inducers. Their binding stimulates caspase-8 and -9 activation, which then trigger executioner caspase activation including caspase-3, caspase-6, and caspase-7 causing cellular apoptosis and the progression of DM-induced vascular disorder [94,95].

**Figure 2:**
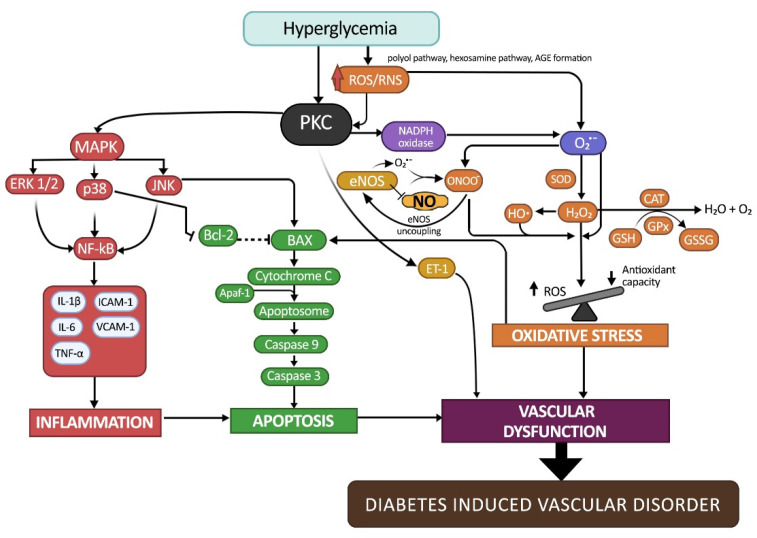
A summary of the genes implicated in DM-induced vascular disorder development. Hyperglycemia resulting from insulin dysregulation induces overproduction of ROS and RNS, generating oxidative stress in the vascular cell. PKC pathways induce inflammation and apoptosis via MAPK pathways. Uncontrolled cellular deaths, as well as oxidative stress, lead to cardiac vascular dysfunction and progress to DM-induced vascular disorder.

The intrinsic pathway is characterized by the dysfunction of mitochondria. In an intrinsic pathway, anti-apoptotic proteins such as Bcl-2 and pro-apoptotic proteins such as Bax and Bak form a pore on the mitochondrial membrane that reaches across both the inner and outer membranes. In rat aortic studies, hyperglycemic conditions diminish Bcl-2 protein expression and enhanced Bax and caspase-3 protein expression [84]. In the mitochondrial membranes, the interaction between pro-apoptotic and anti-apoptotic proteins causes the generation of permeability transition pores. This allows mitochondrial cytochrome C release through these pores to invade cytosolic compartments, then interact with apoptosis protease activating factor-1 to become the apoptosome complex. Apoptosomes are responsible for the activation and breakage of executioner caspases, which lead to apoptosis [96]. Endothelial cell apoptosis is particularly prominent in the development of DM and vascular diseases such as atherosclerosis [97], nephropathy [98], and retinopathy [42]. Figure 2 illustrates the pathogenesis of DM-induced vascular disorder that is caused by oxidative stress, inflammation, and apoptosis.

## 5. Polyphenol

**Figure 3:**
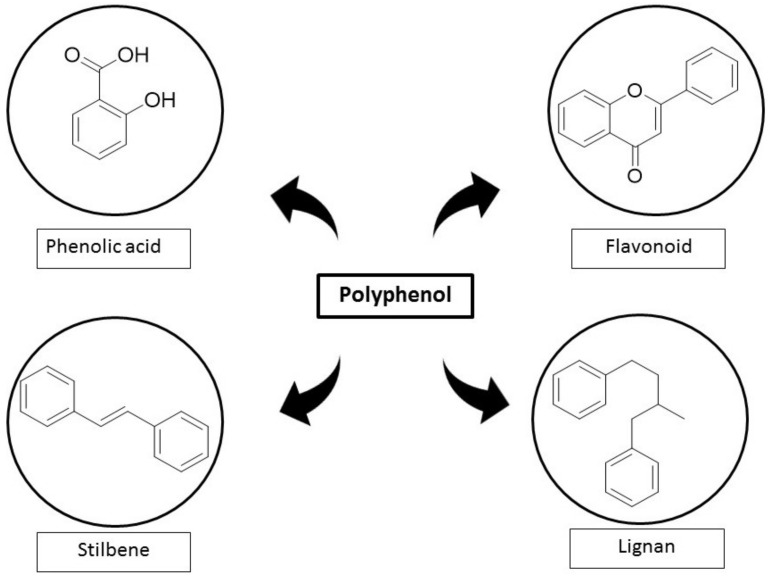
The different types of polyphenol classes’ chemical structure including phenolic acid, flavonoid, stilbene and lignan.

**Figure 4:**
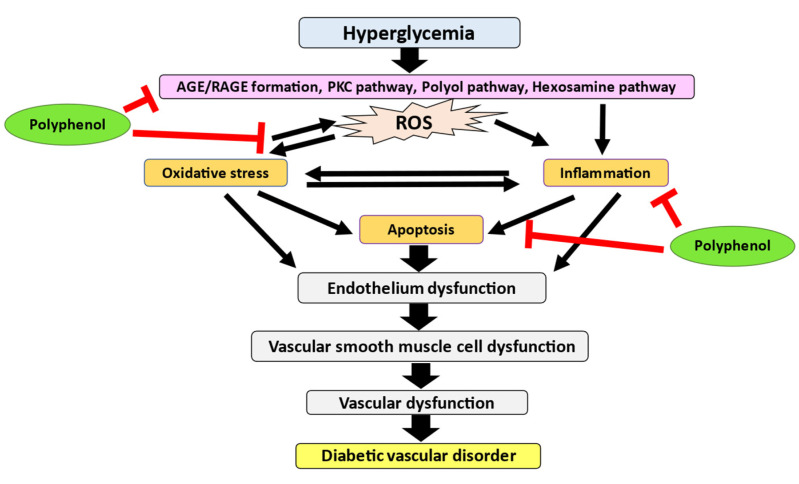
The capacity of polyphenols to resist DM-induced oxidative stress, inflammation, and apoptosis, all of which play critical roles in the structural and functional abnormalities in diabetic vascular disorder, indicates that they may alleviate the diabetic vascular disorder.

Nutritional value from natural food or plants has been widely recognized as a treatment as well as a prevention of many diseases. One of the chemical constituents that can be found abundantly are polyphenols which promote health and impede various types of chronic diseases including DM-induced vascular disorder (Table 1). Polyphenols are phytochemicals that are synthesized by plants with one aromatic ring and a hydroxyl group [99]. They are known to be abundant with antioxidants in the diet, as they are found in various plants such as fruits, vegetables, and nuts. There are four major classes of polyphenols including phenolic acids, flavonoids, stilbenes, and lignans (Figure 3) [100]. Polyphenols have ignited a lot of interest in the scientific community because of their health benefits, especially for metabolic disorders like DM [101]. Since DM is a multifactorial disease, finding alternative therapy that can target many mechanisms is critical. Polyphenols have the ability to modulate signaling pathways such as oxidative stress, inflammation, and apoptosis since they are rich in antioxidant capacity [102,103]. Hence, polyphenols have great potential in ameliorating DM-induced vascular disorder (Figure 4).

## 6. The Role of Polyphenol in Modulating Diabetes-Induced Vascular Disorder

Polyphenols have previously been recognized to be a powerful free radical scavenger, particularly acting against the increased formation of ROS, which has been associated with vascular disease. Uncontrolled ROS generation is known to disrupt vascular tone, which is driven by decreased NO bioavailability, resulting in dysfunctional endothelium-dependent vasodilation [104]. There is now a growing body of evidence that polyphenolic-rich plants are able to protect against vascular dysfunction.

In a previous study, Furuuchi and colleagues [105] found that polyphenols in boysenberry could inhibit endothelial dysfunction and help to restore vascular homeostasis by limiting ROS generation and boosting NO production in the aorta, as well as diminishing p53 and eNOS monomer levels. Furthermore, polyphenol-rich diets have been found to protect vascular cells from oxidative damage, elicit vasorelaxant effects via the SIRT-AMPK pathway, and improve endothelial activity [106].

### 6.1. Phenolic Acids

The term phenolic acids refers to any phenolic substance with a single carboxylic acid group [107]. There are two types of phenolic acids: hydroxybenzoic acids and hydroxycinnamic acids. Benzoic acids (C6-C1) and cinnamic acids (C6-C3) are two types of phenolic acids that contain seven and nine carbon atoms, respectively [108]. Phenolic acids are found in a variety of nuts and fruits, including raspberries, grapes, strawberries, walnuts, cranberries, roselle, and blackcurrants, among others [107]. These are secondary metabolites produced by the shikimate/chorismate pathway from phenylalanine and tyrosine. However, these constituents are mostly encountered as hydroxybenzoic acids (gallic acid, salicylic acid, protocatechuic acid, ellagic acid, and gentisic acid) and hydroxycinnamic acids (such as p-coumaric acid, caffeic acid, ferulic acid, chlorogenic acid, and sinapic acid) or in conjugated forms [13]. Increasing evidence suggests that consuming phenolic acids can help prevent DM-induced vascular disorder by tackling oxidative stress, inflammation, fibrosis, and apoptosis.

Chlorogenic acid is a phenolic acid commonly found in coffee, strawberries, and sunflowers [109]. Wang and colleagues [18] found that supplementation of chlorogenic acid improved endothelial deterioration in diabetic mice via activating the nuclear factor erythroid 2–related factor 2 (Nrf2) anti-oxidative pathway. The upregulation of Nrf2 induced by chlorogenic acid lowered the expression of NADPH oxidase subunits P22phox, P47phox, and P67phox, and also the formation of nitrotyrosine, a biomarker of ONOO^−^ mediated nitration that plays a vital role in vascular damage under high blood glucose levels. Furthermore, in an in vivo experimental design, chlorogenic acid was found to reduce superoxide levels while maintaining NO levels. In mice, the treatment with chlorogenic acid improved endothelial function by maintaining ACh-induced aortic relaxation.

Our earlier findings indicate that roselle, which contains significant amounts of phenolic acids including hibiscus acid and protocatechuic acid, has antioxidant capabilities, lowering malondialdehyde (MDA) and advanced oxidation protein products (AOPP) levels while increasing GSH generation [110]. This could be accomplished by reversing endothelial dysfunction and oxidative vascular damage by regulating NADPH oxidase activity and eNOS uncoupling. Roselle also restored vascular endothelial dysfunction and increased NO bioavailability in insulin-resistant mice by restoring plasma NOX levels and eNOS expression, which reduced superoxide formation [111]. Furthermore, when treated with phenolic acid-rich roselle extract, endogenous antioxidants such as catalase, SOD, and GSH levels were enhanced [19].

Phenolic acid is also important for reducing inflammation. Caffeic acid is one of the phenolic acids that has been shown to alleviate the inflammatory processes induced by hyperglycemia in the ECs. It could act as a possible anti-inflammatory by suppressing C-reactive protein through mechanisms involving NADPH oxidase-dependent oxidative stress, which reduces VCAM-1 and MCP-1 production in diabetic ECs [112]. Besides, caffeic acid inhibits oxidative damage to the endoplasmic reticulum via downregulating RAGE expression. Interestingly, ferulic acid improved the structure of the aortic endothelial wall, lowering triglyceride, total cholesterol, LDL-C, and Ox-LDL levels, boosting the formation of NO and eNOS, and hampering MCP-1, TNFα, and NFκB P65 over-reaction [113]. This implies that ferulic acid can alleviate vascular endothelial dysfunction by regulating inflammatory pathways in DM models.

Likewise, in an in vitro study of human aortic ECs, blueberry metabolites rich in phenolic acids, such as vanillic acid, were found to revive cell-surface glycosaminoglycan and attenuate endothelial inflammatory response by impeding monocyte adhesion, reducing IL8 and VCAM1 expression, and restoring the levels of sulfated glycosaminoglycan under diabetic conditions [114].

### 6.2. Flavonoids

Flavonoids are an imperative group of natural products since they make up one of the largest groups of secondary metabolites found in plants. Flavonoids are bioactive chemicals with one or more aromatic rings containing hydroxyl groups found mostly in dietary plants such as fruits, vegetables, cereals, and drinks consumed daily by humans. Flavonoids feature a basic chemical structure that comprises two aromatic rings bonded by three carbon chains to produce an oxygenated heterocyclic ring. They all have the same chemical structure, which is the C6-C3-C6 backbone [115]. Flavonoids are divided into subclasses such as flavones, flavonols, flavanols, flavanones, isoflavones, and anthocyanins. The numbers and positions of hydroxyl groups in flavonoids’ structure have been linked to antioxidant activity. They protect against oxidant damage by absorbing electrons and forming stable phenoxyl radicals [116].

Moreover, prior studies discovered quercetin, a ubiquitous flavonol exhibiting antioxidative and anti-inflammatory capabilities. Under hyperglycemic circumstances, quercetin boosts antioxidant defense systems by reducing elevated levels of cellular iNOS-derived NO, MDA, and ROS, by scavenging free radicals and restoring the endogenous antioxidant, GSH [117]. Quercetin also promotes eNOS activation, NO generation, and arterial relaxation in diabetic aorta via P13/K/Akt and AMPK signaling, reported by Taguchi [118]. Apart from that, quercetin administration increased NO generation and enhanced ACh-induced vasorelaxation. Additionally, dietary quercetin significantly prevents vascular endothelial dysfunction in diabetic animals by reducing the expression and activity of vascular myeloperoxidase (MPO) [119]. MPO has the unique ability to oxidize calcium ions using H_2_O_2_ and thereby cause vascular damage.

Naringin, a flavanone derived from citrus fruit, has been shown to stimulate Nrf2 activation, a major antioxidant response element (ARE) signaling pathway. As a result, HO-1 expression is upregulated, providing a defense against DM and FFA-induced apoptosis in ECs [120]. It was also discovered that naringin pretreatment is involved in PI3K/Akt or JNK in endothelial HO-1 induction via Nrf2 activation.

Hyperglycemia is widely documented for altering the architecture and function of arteries via various mechanisms, as well as raising the contractile response. However, apigenin, a flavonoid-rich polyphenol, was proven to diminish the maximum contractile response of aortic rings to phenylephrine while enhancing the relaxation degree of aortic rings contracted in response to acetylcholine, indicating that apigenin is a potent antioxidant effect by regulating NO production [121].

Anthocyanin is a type of flavonoid that has been one of nature’s most potent antioxidants as it is capable of lowering oxidants and thereby preventing many chronic diseases, including diabetic vasculopathy, which is linked to oxidative damage, inflammation, and cell death. According to the earlier study, supplementation of blueberry anthocyanin extract effectively diminished hyperglycemia-induced damage by increasing SOD, HO-1, lowering ROS formation and NOX4 expression, and boosting cell vitalities [122]. It also had a vasodilatory effect by boosting NO, eNOS, and PPAR levels while lowering the vasoconstrictors’ angiotensin-converting enzyme (ACE), xanthine oxidase-1 (XO-1), and LDL levels. The bioactivities involved the activation of the PI3K/Akt signaling pathway as well as the breakdown of the PKC pathway.

Interestingly, anthocyanin extracted from sour cherry was capable of preventing the elevation of ROS levels in ECs, showing the anthocyanin’s significant antioxidant action [123]. Similarly, in hyperglycemic conditions, the anthocyanin-rich sour cherry extract was observed to downregulate the proinflammatory cytokines expression such as TNFα, IL-6, IL-8, and IL-1 in in vitro study, as well as promote NOS expression and inhibit ET-1 and endothelin-converting enzyme-1 (ECE-1) expression. In the diabetic aorta, anthocyanin has also been shown to reduce oxidative stress and inflammation by inhibiting IL-6 production via the NFκB signaling pathway, as well as prevent apoptosis by limiting caspase-1 activation [124].

Strawberry anthocyanin has been revealed in a diabetic model to reduce monocyte binding to vessels, lower blood pressure to normal levels, and promote endothelial-dependent vasorelaxation. In fact, strawberry anthocyanin efficiently inhibited the increment of inflammatory mediators such as MCP-1, VCAM1, and ICAM1 as well as E-selectin, NOX2 and NFκB, TNFα in ECs, as well as raised eNOS and Nrf2 expression [125].

### 6.3. Stilbenes

Stilbenes are phenolic compounds found as secondary metabolites in plants and have been categorized as phytoalexins [126]. The stilbene structure is built on the C6-C2-C6 skeleton, which is made up of two aromatic rings bonded by an ethylene bridge. Resveratrol is the most well-known and well-characterized stilbene. More than 400 stilbenes derivatives have been discovered. Their structures span from monomers to octamers, with diverse substituents such as glycosyl, hydroxyl, methyl, or isopropyl groups at various positions [126]. Stilbenes are only found in a few types of foods, such as grapes, red wine, peanuts, and some types of berries in the human diet [127].

Resveratrol is a natural phytoalexin present in numerous plants, foods, and drinks that has a wide range of biological and pharmacological effects through modulating various signaling pathways [128]. Resveratrol has been shown to suppress the pro-atherosclerotic effects of hyperglycemia on ECs by limiting ET-1 and E-selectin mRNA expression and boosting endothelial NO synthase via eNOS/NO signaling, which is triggered by SIRT1 activation [129].

Moreover, resveratrol is important for regulating inflammatory processes. According to a study by Xu et al. 2019, resveratrol reduces the proliferation and migration of VSMCs exposed to high glucose by inactivating NFκB signaling, an effect that is identical to miR-138 inhibitors that promote SIRT1 expression [130]. Furthermore, resveratrol hindered hyperlipidemia and the progression of aortic atherosclerosis lesions in T1DM LDL receptor-deficient mice via activation of AMPK signaling [131]. In DM rat models with coronary heart disease, resveratrol consistently lowered blood glucose levels, and reduced serum triglycerides, glycerides, and inflammation markers, thereby mitigating coronary damage [132]. Resveratrol manifests very powerful relaxant effects on kidney arteries in DM models by enhancing vasodilator effects from NO and increasing the expression of potassium voltage channels [133]. These findings point to resveratrol’s renovascular protection in DM. In a double-blind, randomized, placebo-controlled study, resveratrol supplementation improved arterial stiffness and reduced oxidative stress in T2DM patients, suggesting anti-atherosclerotic effects of resveratrol in clinical trials [134].

Another type of stilbene, piceatannol was able to inhibit monocyte adhesion to the endothelium, scavenge ROS, and reduced ROS production, and NFκB activation which then protected endothelial barrier function under hyperglycemic condition [135]. Apart from that, piceatannol was proven to upregulate the expression of HO-1 through the Nrf2 signaling pathway in the ECs, hence decreasing the production of IL-6 and TNFα, and the formation of ROS via NFκB activation. Piceatannol also successfully counteracted the inhibitory effects of palmitate acid on IRS-1 and eNOS phosphorylation driven by hyperglycemia [136].

### 6.4. Lignans

Lignans are a bioactive compound that has caught the interest of food chemists and nutritionists in recent years. They are a group of secondary metabolites that are generated from the joining of two phenylpropanoid C6-C3 units at the β and β’ carbon and can be bonded to additional ether, lactone, or carbon bonds. Their chemical structure is similar to that of 1,4-diarylbutane [137]. Their structure and biological activity cover a wide spectrum. Lignans are generated from the shikimic acid pathway [138]. The commonly reported compounds are secoisolariciresinol, lariciresinol, matairesinol, pinoresinol, medioresinol, and syringaresinol. Lignan has long been recognized for its role in disease prevention and promotion. Several studies have demonstrated the ability of lignans to protect against the development of numerous diseases, including DM-related vascular disorder.

In an earlier study, flaxseed supplementation that is rich in secoisolariciresinol in DM rats ameliorates DM-induced vascular reactivity and endothelial-dependent relaxation by enhancing NO bioavailability and modulating prostaglandin dependent pathways in the aorta [139]. This suggests that secoisolariciresinol protects the vasculature with antioxidant properties by scavenging ROS in diabetic vasculopathy. Apart from that, sesamin, a lignan in sesame seed, is able to diminish the elevated MDA and SOD which then elucidates the lipid peroxidation and oxidative stress, and preserve vascular function in aortic ring under hyperglycemic conditions [140].

Another type of lignan, honokiol, is found abundantly in magnolia genus plants and has been demonstrated to reverse hyperglycemic conditions by targeting oxidative stress and apoptosis [141]. Honokiol is able to reduce the ROS, SOD, and MDA levels as well as downregulate the expression of CHOP, GRP78, p-PERK, p-IRE1α and cleaved caspase-3 and SIRT1, which preserves the cell viability. Honokiol-activated SIRT1 promotes and then interacts with Akt, consequently activating Akt activity which later inhibits apoptosis in DM-induced endothelial dysfunction.

**Table 1:** Summary of the protective effect of polyphenols in ameliorating diabetes-induced vascular disorder.

Classes	Type	Study Design	Dose	Example of the Effects/Associated Pathways	Reference
Phenolic acid	Chlorogenic acid	*In vitro & in vivo*	10 μM & 0.02%	Ameliorates endothelial dysfunction via activation of Nrf2 anti-oxidative pathway	[18]
	Hibiscus acid Protocatechuic acid	*In vivo*	100 mg/kg	Exerts protective effects by enhancing the levels of CAT, SOD, GSH, HDL-C as well as reducing the levels of MDA and LDL-C	[19]
	Hibiscus acid Protocatechuic acid	*In vivo*	100 mg/kg	Improves dyslipidemia, reverses oxidative stress by decreasing MDA and AOPP, as well as increasing GSH levels	[110]
	Caffeic acid	*In vitro*	10 μM	Suppresses production of CRP, VCAM-1, and MCP-1 in glycated LDL by downregulating RAGE expression and oxidative damage in endothelial cells	[112]
	Ferulic acid	*In vivo*	50 mg/kg	Restores the architecture of the aortic endothelium wall, ameliorating the increase of HbAlc, TG, TC, LDL-C, and Ox-LDL, stimulating the secretion of NO and eNOS, and hampering activation of MCP-1, TNFα, and NFκB P65 to normal levels	[113]
	Vanillic acid	*In vitro*	75 nM	Attenuates endothelial inflammation of human aortic endothelial cells by suppressing monocyte binding and reduces IL8 and VCAM1 expression as well as restoring the levels of sulfated glycosaminoglycan	[114]
Flavonoid	Quercetin	*In vitro*	0, 0.1, 1, 10, 20, 50, 100, 200, 400 μM	Increases cell survival of HUVEC cells, decreases total level of oxidative stress, increases activity of GSH	[117]
	Quercetin	*In vivo*	10–9 to 10–5 µM	Escalates the phosphorylation of Akt and eNOS, PI3K and AMPK expression is suppressed as well as NO production, and AMPK phosphorylation	[118]
	Quercetin	*In vitro & in vivo*	5−20 μM & 3.5 mg	Attenuates HOCl-caused endothelial dysfunction by limiting MPO/H2O2 dependent HOCl production, suppresses MPO activity and expression	[119]
	Naringenin	*In vitro*	0–100 μM	Enhances HO-1 expression, activation of P13K/Akt, ERK, JNK, stimulate Nrf2, reduces FFA-induced cell apoptosis	[120]
	Apigenin	*In vivo*	10 mg/kg	Suppresses contractile response of aorta	[121]
	Anthocyanin	*In vitro*	5 μ/mL	Enhances endogenous antioxidant SOD, HO-1, lowering ROS generation and NOX4 expression, increasing NO, eNOS, and PPAR, stimulates PI3K/Akt signaling pathway and the breakdown of PKC pathway	[122]
	Anthocyanin	*In vitro*	50 μL/mL	Alleviates oxidative damage and inflammation via the inhibition of NFκB expression as well as suppressing apoptosis by decreasing activation of caspase-1	[124]
	Anthocyanin	*In vivo & in vitro*	2.35% freeze-dried strawberry supplemented diet	Diminishes monocyte binding to the vessel wall, downregulating the expression of MCP-1/JE, KC, VCAM-1, IκKβ, and NOX2 are reduced	[125]
Stilbene	Resveratrol	*In vitro*	1 μmol/L	Upregulation of SIRT1 and increase in the generation of NO and eNOS, which counteracts other pro-atherosclerotic effects of hyperglycemia by upregulation of ET-1	[129]
	Resveratrol	*In vivo*	10 mg/kg/day	Reduces inflammatory factors including TNFα, IL-6, IL-8, intracellular adhesion molecule 1, MCP-1, and downregulates the expression of signaling pathway TLR4/MyD88/ NFκB	[132]
	Resveratrol	*Ex vivo*	1–100 μM	Manifests potent relaxant effects on renal artery mediated by NO mechanism and potassium channels	[133]
	Resveratrol	Clinical	100 mg	Improves arterial stiffness and oxidative stress	[134]
	Piceatannol	*In vitro*	10 μM	Decreases the monocyte adhesion to the endothelium, prevents the increase in ICAM-1 protein level, scavenges ROS, and reduces NFκB activation	[135]
	Piceatannol	*In vitro*	20 μM	Elevates the expression of HO-1 accompanied by HO activity, increases Nrf2 expression, suppresses the secretion of TNFα, IL-6, ROS generation, decreases phosphorylation p65, and increases the phosphorylation of eNOS which restores NO production	[136]
Lignan	Secoisolariciresinol	*In vivo*	0.714 g/kg	Improves vascular reactivity by increasing NO bioavailability and modulating PG dependent mechanisms	[139]
	Sesamin	*In vivo*	10 & 20 mg/kg	Improves oxidative stress status by reversing the increased MDA and elevating the activity of SOD, preventing the functional changes of vascular reactivity through NO and PG pathway	[140]
	Honokiol	*In vitro*	5, 20, 80 μmol/L	Reverses the effect of apoptosis, ROS and MDA levels, and the expressions of CHOP, GRP78, p-PERK, p-IRE1α, and cleaves caspase-3, as well as restores the inhibitory effect of cell viability, SOD level, and SIRT1 mechanisms to normal levels	[141]

## 7. Conclusions

There is a major epidemic of DM-induced vascular dysfunction and its associated complications that currently requires therapeutical intervention to reduce the elevated morbidity and mortality worldwide. It is clear that hyperglycemia perpetuation can lead to DM-induced vascular disorder via oxidative stress, inflammation, and apoptosis mechanisms. The research findings of the above studies suggest that polyphenols (phenolic acid, flavonoid, stilbene, and lignan) may provide therapeutic benefits for vascular dysfunction in DM. Together, these findings provide compelling support for the identification of more potent pharmacological agents from natural products to add to the arsenal of treatments for DM-induced vascular injury, especially atherosclerosis and coronary heart disease. Targeting the important associated genes that modulate the oxidative stress, inflammation, and apoptosis pathways that can lead to DM-induced vascular disorder is crucial for effective vascular management.

## Data Availability

No new data were created or analyzed in this study. Data sharing is not applicable to this article.

## Abbreviations

AGEs	Advanced glycation end products
AMPK	5′ adenosine monophosphate-activated protein kinase
ATP	Adenosine triphosphate
BH4	Tetrahydrobiopterin
CVD	Cardiovascular diseases
DAG	Diacylglycerol
DM	Diabetes mellitus
DNA	Deoxyribonucleic acid
ECs	Endothelial cells
eNOS	Endothelial nitric oxide synthase
ERK	Extracellular signal-regulated kinase
ETC	Electron transport chain
FADH_2_	Flavin adenine dinucleotide
FFA	Free fatty acid
GSH	Glutathione
HDL	High density lipoprotein
HIF-1α	Hypoxia-induced factor 1α
HO-1	Heme oxygenase 1
ICAM	Intercellular adhesion molecule
IL	Interleukin
LDL	Low density lipoprotein
LP	Lipoprotein
MAPK	Mitogen-activated protein kinase
MCP	Monocyte chemoattractant protein
MnSOD	Manganese superoxide dismutase
MPO	Myeloperoxidase
NADH	Nicotinamide adenine dinucleotide
NADPH	Reduced nicotinamide adenine dinucleotide phosphate
NFκB	Nuclear factor kappa light chain enhancer of activated B cells
NO	Nitric oxide
NOX	NADPH oxidase
Nrf2	Nuclear factor-erythroid factor 2
ONOO^−^	Peroxynitrite
P13K	Phosphoinositide 3-kinase
PAD	Peripheral artery disease
PKC	Protein kinase C
RAGE	Advanced glycation end products receptor
ROS	Reactive Oxygen Species
SIRT	Sirtuin
SOD	Superoxide dismutase
TCA	Tricarboxylic acid
TNFα	Tumor necrosis factor α
VCAM	Vascular cell adhesion molecule
VSMC	Vascular smooth muscle cell
